# Capsular polysaccharide determines the serotyping of *Riemerella anatipestifer*

**DOI:** 10.1128/spectrum.01804-23

**Published:** 2023-10-12

**Authors:** Yanling Liu, Shuxin Luo, Zhishuang Yang, Mingshu Wang, Renyong Jia, Shun Chen, Mafeng Liu, Xinxin Zhao, Qiao Yang, Ying Wu, Shaqiu Zhang, Juan Huang, Xumin Ou, Sai Mao, Qun Gao, Di Sun, Bin Tian, Anchun Cheng, Dekang Zhu

**Affiliations:** 1 Research Center of Avian Diseases, College of Veterinary Medicine, Sichuan Agricultural University, Chengdu, Sichuan, China; 2 Key Laboratory of Animal Disease and Human Health of Sichuan Province, Chengdu, Sichuan, China; 3 International Joint Research Center for Animal Disease Prevention and Control of Sichuan Province, Chengdu, Sichuan, China; Texas A&M University, College Station, Texas, USA

**Keywords:** *Riemerella anatipestifer*, capsule, serotyping, capsular polysaccharide, lipopolysaccharide

## Abstract

*Riemerella anatipestifer* (*R. anatipestifer*) is a Gram-negative pathogen that causes significant economic losses to the farming industry. The polysaccharides that determine serotype are well understood in other bacteria; they have not yet been fully characterized in *R. anatipestifer*. In this study, we unexpectedly discovered a strain, RCAD0392, that is capable of cross-agglutination. Whole genome sequencing revealed base mutations disrupting a gene on its capsular polysaccharide synthesis gene cluster. By constructing homologous gene deletion mutants in CH-2, we demonstrated that deletion of this gene leads to altered serological phenotypes. India ink and transmission electron microscopy experiments revealed the disappearance of capsule on the surface of the bacteria, indicating the association of the gene with capsule synthesis. In addition, agar-gel precipitin tests showed that lipopolysaccharide binds to antisera of multiple serotypes, while the capsular polysaccharide only binds to the corresponding antisera. This suggests that capsular polysaccharide is the specific antigen that determines the serotype of *R. anatipestifer*.

**IMPORTANCE:**

*Riemerella anatipestifer* (*R. anatipestifer*) is one of the most important veterinary pathogens with at least 21 serotypes. However, the exact polysaccharide(s) that determine *R. anatipestifer* serotype is still unknown. This study has provided a preliminary exploration of the relationship between capsular polysaccharides and serotyping in *R. anatipestifer* and suggests possible directions for further investigation of the genetic basis of serotypes in this bacterium.

## INTRODUCTION

*Riemerella anatipestifer* (*R. anatipestifer*) is a Gram-negative bacterium that infects ducks, geese, turkeys, and other avian species, causing severe septicemia and serositis. *R. anatipestifer* infection has caused significant economic losses in the duck industry worldwide. *R. anatipestifer* has at least 21 serovars (1), and the protective efficacy of vaccines has been limited due to the absence of cross-protection between serovars (2).

Based on the antigenic differences of capsular polysaccharide (CPS) and lipopolysaccharide (LPS), a bacterial species can be classified into different serovars by serotyping. At present, the serotyping of CPS and O-antigen have been widely used in the identification and typing of a variety of bacteria (3, 4). According to the antigenicity of capsular polysaccharides, there were more than 90 serovars of *Streptococcus pneumoniae* (5), 35 serovars of *Streptococcus suis* (6), 5 serovars of *Pasteurella multocida* (7), and 82 serovars of *Klebsiella pneumoniae* (8). According to the antigenicity of lipopolysaccharide, there were 16 serovars of *Pasteurella multocida* (9, 10) and 9 serovars of *Klebsiella pneumoniae* (3). Currently, serotyping of *R. anatipestifer* is based on surface polysaccharide antigens (11). Although there have been several studies focusing on genes related to LPS (12 – 14) and CPS (15) synthesis in *R. anatipestifer*, the molecular determinants of *R. anatipestifer* serotyping have not been elucidated.

In this study, we focused on the molecular basis of RA serotyping. During routine surveillance, we identified a clinical isolate, RCAD0392, that cross-agglutinated with several antisera of different serotypes. Whole genome sequencing revealed that a base deletion disrupted the *D1J34_RS08130* gene (corresponding to *G148_RS04320* of RA CH-2) on the CPS synthesis gene cluster. Further phenotypic testing using India ink and transmission electron microscopy confirms that the CPS is the specific antigen that likely distinguishes serovars of *R. anatipestifer*. Our study provides a basis for serotyping of *R. anatipestifer* using the CPS genotyping system and reveals the molecular basis of *R. anatipestifer* serotyping.

## MATERIALS AND METHODS

### Bacterial strains and growth conditions

The bacterial strains, plasmids and primers used in this study are listed in Tables 1 and 2, respectively. The *R. anatipestifer* strains were grown in tryptic soy broth (TSB), GC broth (GCB) or tryptic soy agar (TSA) (Oxoid Ltd., Basingstoke, UK) at 37°C supplemented in 5% CO_2_. *Escherichia coli* strains were cultured in Luria Bertani (LB) broth at 37°C. Antibiotics were added, when necessary, to a final concentration of 40 µg/mL for erythromycin (Erm) and 100µg/mL for ampicillin. All antibiotics were obtained from Dalian Meilun Biotech Co., Ltd. (Dalian, China). The bacterial growth was monitored by measuring absorbance at 600 nM (OD_600_).

**TABLE 1:** The strains used in this study^*a*^

Strain or plasmid	Descriptions	Resources or references
*R. anatipestifer* RCAD0392	Wild strain	Laboratory collection
*R. anatipestifer* CH-2	Wild strain	Laboratory collection
CH-2 Δ*G148_RS04320*	*G148_RS04320* deletion mutant of CH-2 strain	This study
c*G148_RS04320*	*G148_RS04320* complemented mutant of CH-2 strain	This study
*R. anatipestifer* CCUG 18373	Serovar 1	CCUG
*R. anatipestifer* CCUG 25001	Serovar 2	CCUG
*R. anatipestifer* CCUG 25002	Serovar 3	CCUG
*R. anatipestifer* CCUG 25004	Serovar 5	CCUG
*R. anatipestifer* CCUG 25005	Serovar 6	CCUG
*R. anatipestifer* CCUG 25054	Serovar 8	CCUG
*R. anatipestifer* CCUG 25006	Serovar 9	CCUG
*R. anatipestifer* CCUG 25008	Serovar 10	CCUG
*R. anatipestifer* CCUG 25013	Serovar 11	CCUG
*R. anatipestifer* CCUG 25055	Serovar 12	CCUG
*R. anatipestifer* CCUG 25012	Serovar 13	CCUG

^*a*^ CCUG, Culture Collection of the University of Gothenburg.

**TABLE 2:** Primers used in this study

Primers	Sequence (5’−3’)	Source
G148_RS04320 UPP1	CTGGGGGCGAAAAAAAATTGCC	This study
G148_RS04320 UPP2	CCTGCCTGCGTGTAGCTGAAAAATACCAACCTAAAAACAAAGAGAACCG	This study
ErmP1	CGGTTCTCTTTGTTTTTAGGTTGGTATTTTTCAGCTACACGCAGGCAGG	This study
ErmP2	GCAAATATACAAATTTAAAAGCATCTACGAAGGATGAAATTTTTCAGG	This study
G148_RS04320 DOWNP1	CCTGAAAAATTTCATCCTTCGTAGATGCTTTTAAATTTGTATATTTGC	This study
G148_RS04320 DOWNP2	CAAACATTGCTGCTGATTGTCTC	This study
G148_RS04320 P1	GCGACAAATTAGTATGGTTG	This study
G148_RS04320 P2	CTACTTATCTAAAGACTCAAACACA	This study
cG148_RS04320 P1	CATGCCATGGGCGACAAATTAGTATGGT	This study
cG148_RS04320 P2	CCGCTCGAGCTACTTATCTAAAGACTCA	This study

### Whole genome sequencing and bioinformatics analyses

The whole-genome DNA was extracted by the Bacterial Genome Extraction Kit (Tiangen Biochemical Technology Co., Ltd, Beijing, China) according to the manufacturer’s instructions. Paired-end and PacBio libraries were constructed using the TruSeq DNA PCR-Free Sample Preparation Kit (Illumina, USA) and PacBio SMRTbell Template Prep Kit (Pacific Biosciences) following the manufacturer’s protocol. These libraries were sequenced using paired-end Illumina (Illumina HiSeq 2500) and PacBio (PacBio RS II platform). The genome was assembled by CANU (version 1.5) (16) with PacBio reads, and corrected twice using pilon (version 1.22) (17) with Illumina reads. The annotation was performed using the NCBI Prokaryotic Genome Annotation Pipeline (PGAP) (18). To investigate the genomic variation of RCAD0392 against RACH-2, snippy version 4.6.0 (https://github.com/tseemann/snippy, default parameters) was used to map the reads to RA-CH-2 genome (Refseq accession: NC_020125.1) and call variants. Large structural variations were detected using the breseq split-read analysis tool with default parameters (19).

Based on the description of the previous study (20), we predicted the capsular gene clusters. Briefly, we retrieved entries involving capsular polysaccharide synthesis from the Pfam database by searching for keywords (https://www.ebi.ac.uk/interpro/entry/pfam/, Table 3). Next, we searched for these pfam profiles in the *R. anatipestifer* genome using the HMMER 3.3.2 (e-value cutoff <1e-10). This allowed a CPS gene cluster to be clearly identified in the genome.

**TABLE 3:** Accession and description of all protein profiles searched in *R. anatipestifer* genomes to identify putative CPS synthesis clusters

Pfam accession	Protein name	Pfam name	Description	Class of Pfam
PF00005.30	KpsT	ABC_tran	ABC transporter	Capsule synthesis
PF01061.27	KpsM	ABC2_membrane	ABC-2 type transporter	Capsule synthesis
PF05159.17		Capsule_synth	Capsule polysaccharide biosynthesis protein	Capsule synthesis
PF04932.18	Wzy	Wzy_C	Polymerase	Capsule synthesis
PF13425.9	Wzy	O-antigen_lig	Polymerase	Capsule synthesis
PF13440.9	Wzx	Polysacc_synt_3	Flippase	Capsule synthesis
PF14667.9	Wzx	Polysacc_synt_C	Flippase	Capsule synthesis
PF02563.19	Wza (OMP)	Poly_export	Outer membrane export protein	Capsule export
PF01451.24	Wzb	LMWPc	Low molecular weight phosphotyrosine protein phosphatase	Capsule export
PF02706.18	Wzc (IMP)	Wzz	Chain length regulator	Capsule export
PF13614.9	Wzc (IMP)	AAA31	Translocation of macromolecules	Capsule export
PF00535.29	Glycosyl transferase	Glycos_transf_2	Glycosyl transferase family 2	Sugar modifying-enzymes
PF01370.24	Epimerase	Epimerase	NAD dependent epimerase/dehydratase family	Sugar modifying-enzymes
PF16363.8	GDP-mannose 4,6 dehydratase	GDP_Man_Dehyd	GDP-mannose 4,6 dehydratase	Sugar modifying-enzymes
PF00583.28	Acetyltransferase	Acetyltransf_1	Acetyltransferase (GNAT) family	Sugar modifying-enzymes
PF02397.19	Bacterial sugar transferase	Bac_transf	Bacterial sugar transferase	Sugar modifying-enzymes
PF00483.26	Nucleotidyl transferase	NTP_transferase	Nucleotidyl transferase	Sugar modifying-enzymes

Genome sequence and raw reads of RCAD0392 are available in the National Center for Biotechnology Information (NCBI) database with BioProject no. PRJNA487674.

### Construction of CH-2 Δ*G148_RS04320* and the complemented strain

Gene knockout was performed as previously described (21). Briefly, primers *G148_RS04320* UP P1/P2 and *G148_RS04320* DOWN P1/P2 (Table 2) were used to amplify the upstream homologous arm fragment and the downstream homologous arm fragment of the RA CH-2 *G148_RS04320* gene, respectively. Primers ERM P1/P2 (Table 2) were used to amplify the *erm* fragment from the genome of RA CH-1. Three PCR fragments UED (*G148_RS04320* upstream, the *erm* cassette, and *G148_RS04320* downstream) were amplified using the overlap PCR method. The RA CH-2 strains were grown in GCB medium at 37°C and their bacterial density was adjusted to an OD_600_ of 1. Three hundred microliters of the bacterial suspension was placed inside a 1.5 mL sterilized tube, supplemented with the UED fragment (1.2 µg), and incubated for 30 minutes at 37°C. After incubating for 3 hours at 37°C, 300 µL of the mixture were plated onto erythromycin-supplemented plates and incubated overnight at 37°C. Single colonies grown on erythromycin plates were isolated and screened using PCR.

For gene complementation, the *G148_RS04320* gene was amplified using primers c*G148_RS04320* P1/P2 (Table 1). After purification, PCR products were digested with *Nco*I and *Xho*I endonuclease (Takara Biology Co., Ltd, Dalian, China), and then ligated into the shuttle plasmid pLMF03 (22) that was digested with the same endonuclease. The recombinant plasmid pLMF03::*G148_RS04320* was transformed into competent *Escherichia coli* DH5α cells, which were spread on LB plate supplemented with ampicillin, cultured and screened. Subsequently, the recombinant plasmid was transformed into CH-2 Δ*G148_RS04320* by natural transformation. The recombinant colonies grown on the erythromycin and ampicillin plate were isolated and screened, and the correct ones were preserved.

### Capsule staining

To identify the *R. anatipestifer* capsule, we performed negative staining with India ink and observed under light microscopy, following the previously described protocol (23). Briefly, the colony was mixed on a glass slide with a drop of India ink and spread thinly over the whole slide. After drying the slide under air, we saturated it with 1% crystal violet for 2 minutes, washed gently with deionized water, and then dried naturally.

### Transmission electron microscopy (TEM)

To prepare for TEM analysis, the overnight bacterial culture was collected and washed twice using phosphate-buffered saline (PBS). The pellet was fixed with 2.5% glutaraldehyde for 2 hours followed by resuspension, and addition of suspension onto a copper grid covered with a formvar membrane. Then, the formvar membrane was stained with 20 g/L phosphotungstic acid for 1 minute. Samples were observed and analyzed by transmission electron microscope using a JEOL JEM-2500SE instrument (EOL Co., Ltd, Tokyo, Japan).

### Polysaccharide extraction

The extraction and purification of CPS was performed according to the previous description (24). Briefly, the overnight bacterial cultures were diluted to OD_600_ = 0.65 and centrifuged at 9,600 × *g* for 5 minutes. The supernatant was removed and the pellet was resuspended in 4 mL of lysis buffer containing 60 mM Tris (pH 8), 10 mM MgCl_2_, 50 µM CaCl_2_, and incubated at 37°C for 1 hour followed by three freeze-thaw cycles at −80/37°C. DNase and RNase were added to the sample, which was then incubated at 37°C for 30 minutes. Sodium dodecyl sulfate (SDS) was added to the sample and incubated at 37°C for an additional 30 minutes. After incubation, the sample was heated at 100°C for 10 minutes. Proteinase K was added, and the mixture was incubated at 60°C for 1 hour. Then, the mixture was centrifuged at 9,600 × *g* for 5 minutes and the supernatant was collected. Ethanol was added at three times the volume of the supernatant and incubated at −20°C overnight. The material was then centrifuged at 6,200 × *g* for 45 minutes at 4°C and the supernatant was discarded. The precipitate was dried at room temperature, resuspended in PBS, and subjected to ultracentrifugation (Thermo Fisher Scientific Co., Ltd, USA) at 100,000 × *g* for 2 hours. The supernatant containing the CPS was aspirated.

The extraction and purification of LPS were performed according to the previous description (25). Briefly, the overnight bacterial cultures were harvested and washed three times with 1 mL of PBS. The bacterial suspension was adjusted to OD_600_ = 1 and then centrifuged. The pellet was then resuspended in 150 µL of lysis buffer containing 60 mM Tris (pH 6.8), 2% SDS, 4% 2-mercaptoethanol, and 10% glycerol. The sample was boiled for 10 minutes. Proteinase K was added, and the mixture was incubated at 60°C for 1 hour.

### SDS-PAGE

CPS samples were separated on a 10% SDS gel at 80 V for 30 minutes and then 120 V for 90 minutes. The gel was stained with Alcian blue to visualize the presence of CPS.

### Agar-gel precipitin test

To study the antigen-antibody interaction, the agar-gel precipitin test was performed using 1% agarose gel. Agar (1 g) was added to 8.5% NaCl (100 mL) and the solution was boiled using a microwave oven. The agar solution was then poured into culture dishes, and the thickness of gel is 2–3 mm. Seven wells were created, with one in the center and six surrounding wells. Standard antisera or mouse sera (20 µL) was added to the central well, CPS or LPS (20 µL) extracted from different strains was added to the surrounding wells. The agar gel plates were then put in a wet box at 37°C for 24 hours under saturated humidity.

### Serological slide agglutination test

Serovars were determined by slide agglutination test using standard serotyping antisera (RIPAC-LABOR GmbH, Potsdam, Germany) against *R. anatipestifer*. In brief, 10 µL of the standard antiserum for *R. anatipestifer* serotyping was mixed with 10 µL bacteria suspension. The reaction was recorded as positive when clumping of bacteria was observed, while a negative reaction in a turbid liquid. When a strain produces agglutination reactions with multiple (more than one) antisera of different serovars, it is considered as cross-agglutination.

### Preparation of anti-CPS positive serum

Six specific-pathogen-free female Kunming mice (Dashuo Science and Technology Co., Ltd., China) aged 6–8 weeks old and weighing 18–22 g, with similar body conditions, were randomly divided into three groups. Groups of two mice were immunized intraperitoneal with the appropriate antigen on day 0 and boosts were administered on days 7 and 14. Immunization groups were as follows: CPS (500 µg/mL); CPS (1 mg/mL). The control mice were immunized with sterile normal saline. Mice were blood collected, 7 days after the last immunization. According to NC3Rs (26), blood samples were obtained through retro-orbital bleeding after anesthesia. All animal experiments were approved by the Animal Ethics Committee of the Sichuan Agricultural University (approval 2022–031).

## RESULTS

### Identification and mutational analysis of the putative CPS synthesis gene cluster

**Fig 1:**
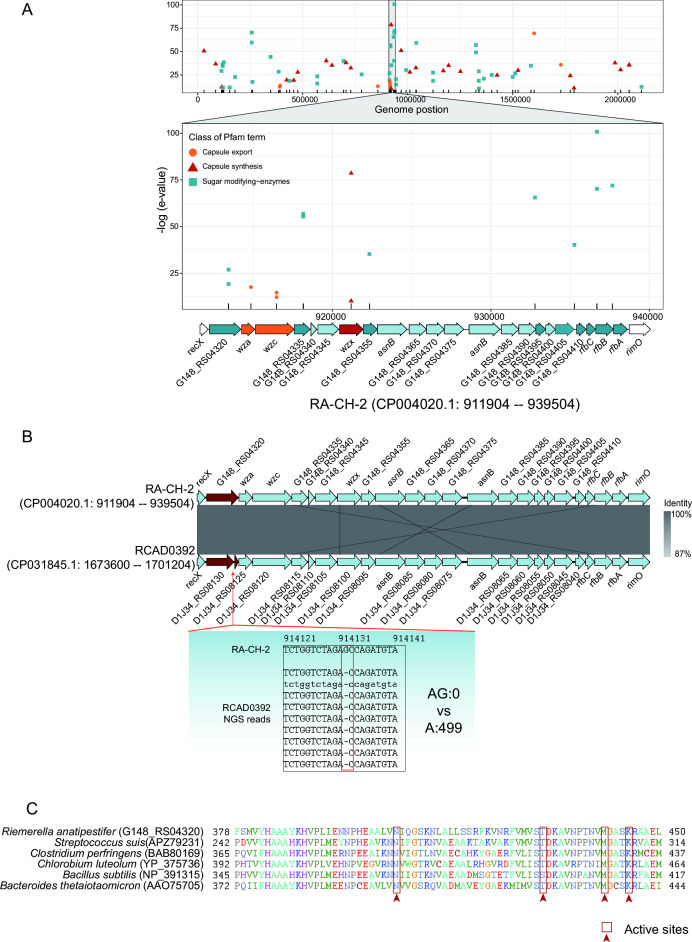
Identification and mutational analysis of the CPS synthesis gene cluster. (**A**) Distribution of CPS synthesis-related genes in the CH-2 genome indicates a putative CPS gene cluster. (**B**) A deletion disrupts the *D1J34_RS08130* gene in the CPS gene cluster of RCAD0392. (**C**) Multiple sequence alignment of the active site region of G148_RS04320 with other CpsE proteins.

We identified a CPS synthesis gene cluster, which contained three well-characterized genes, *wza*, *wzc*, and *wzx*. These genes predicted to encode outer membrane polysaccharide export proteins, protein-tyrosine kinase, and oligosaccharide flippase, respectively (Fig. 1A). Further genomic analysis revealed that the gene cluster locus was conserved across serovars (Fig. S1), with the *recX* gene located upstream and the *rimO* gene located downstream of this gene cluster (Fig. 1).

To explore the genetic basis of cross-agglutination in strain RCAD0392, we focused on the genetic variation in its CPS synthesis gene cluster. Genome-wide variant calling (only 3963 variants with 2852 synonymous mutations, Table S1) indicated considerable genomic identity between RCAD0392 and RA CH-2. We found that a base deletion disrupts the *D1J34_RS08130* gene (corresponding to *G148_RS04320* of RA CH-2, Fig. 1B), which predicted to encode a polysaccharide biosynthesis protein that shared 42.77% identity and 96% coverage with the *Streptococcus suis* CPS synthesis protein CpsE (APZ79231.1) (Fig. 1C). Sequence alignment indicated that G148_RS04320 protein had a conserved active site of CpsE (27 – 29).

### Deletion of G148_RS04320 causes *R. anatipestifer* to cross agglutinate

**Fig 2:**
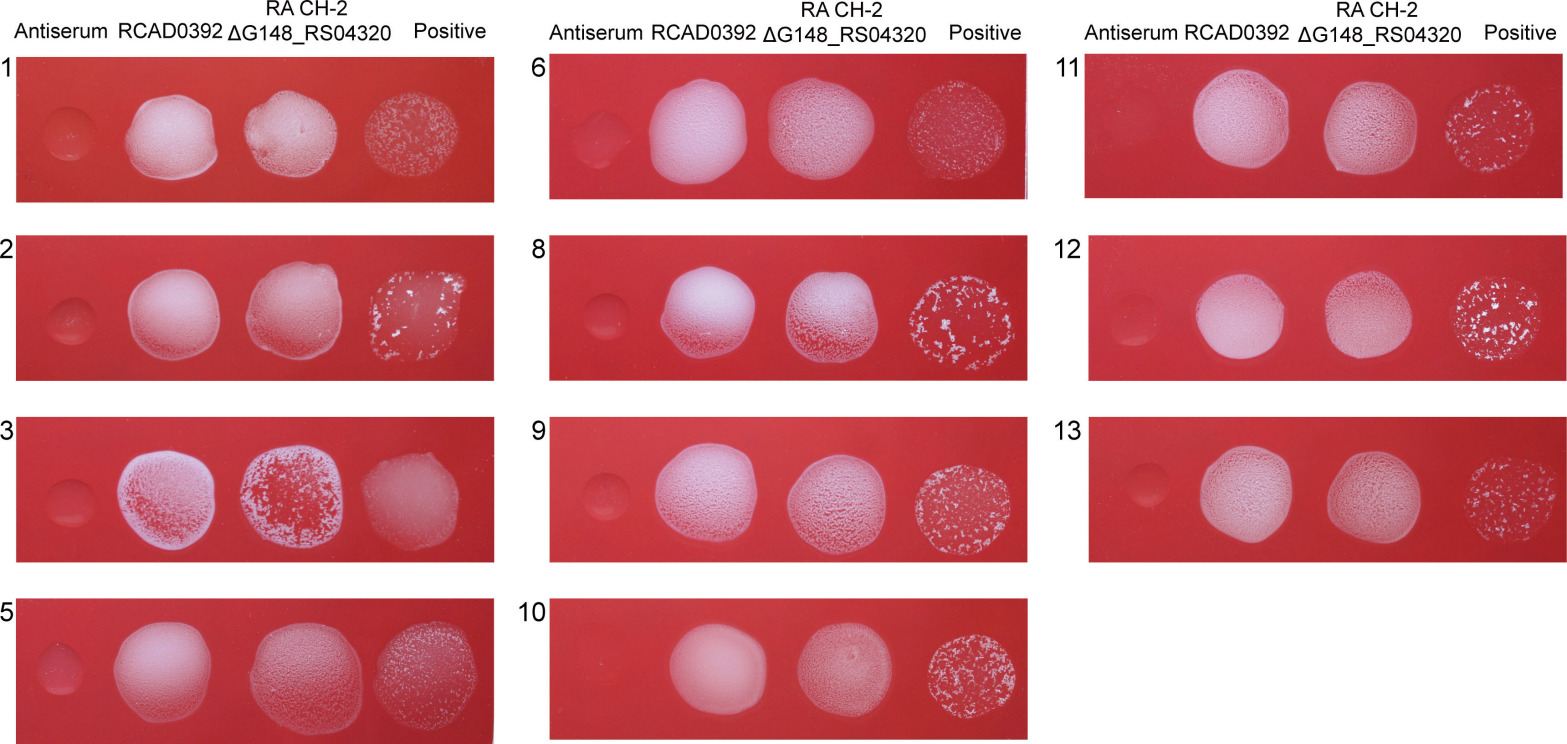
Slide agglutination. Standard antiserum 1–3, 5, 6, and 8–13 for *R. anatipestifer* were mixed with bacterial suspension (RCAD0392, CH-2 ΔG148_RS04320, and positive strains). The numbers in the top left corner of each small picture indicate standard antiserum 1–3, 5, 6, and 8–13. The CH-2 Δ*G148_RS04320* and RCAD0392 strains could agglutinate with all antiserum. The positive strains of types 1–3, 5 ,6, and 8–13 serum were, respectively, CCUG 18373, CCUG 25001, CCUG 25002, CCUG 25004, CCUG 25005, CCUG 25054, CCUG 25006, CCUG 25008, CCUG 25013, CCUG 25055, and CCUG 25012.

To investigate the role of *D1J34_RS08130* gene (corresponding to *G148_RS04320* of RA CH-2) in cross-agglutination of the isolate RCAD0392, we constructed a mutant strain (CH-2Δ*G148_RS04320*) and complementation strain (cCH-2Δ*G148_RS04320*). We successfully constructed both strains, as confirmed by PCR amplification (Fig. S2). Slide agglutination assays showed that the CH-2 Δ*G148_RS04320* strain could agglutinate with multiple antisera (Fig. 2), similar to RCAD0392, whereas the CH-2 and cCH-2 Δ*G148_RS04320* strains only agglutinate with serotype 2 antisera (Fig. S3). These results suggested that deletion of *G148_RS04320* gene could cause changes of the surface polysaccharide antigens and thus affect serological-related phenotypes, leading to cross-agglutination.

### Deletion of G148_RS04320 gene leads to capsule deficiency in *R. anatipestifer*

**Fig 3:**
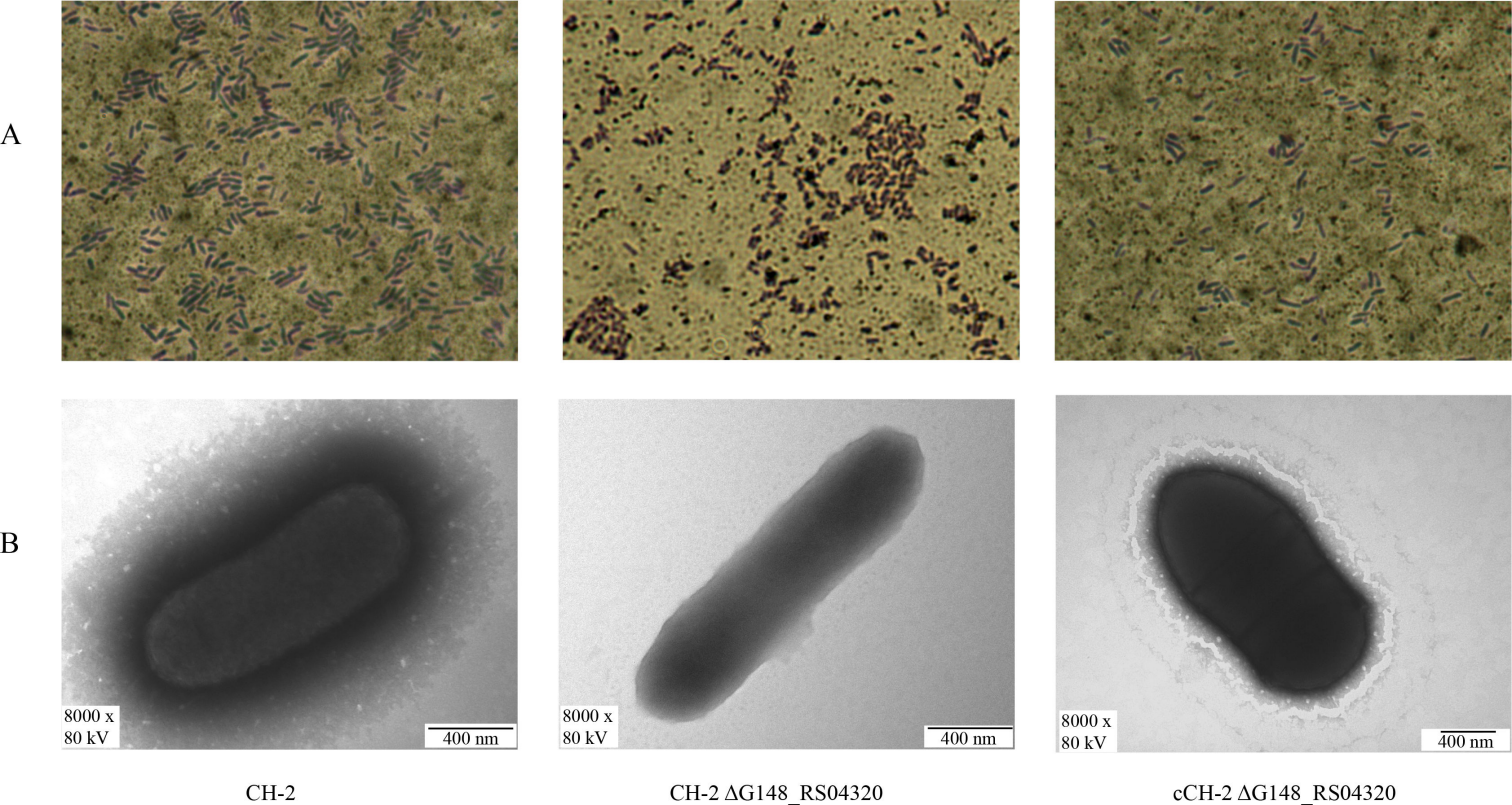
Deletion of *G148_RS04320* results in capsule deficiency. (**A**) Capsule stain. Light microscopy observation of CH-2, CH-2 ΔG148_RS04320, and cCH-2 ΔG148_RS04320 stained by India ink and crystal violet. Capsule was visible as a clear halo surrounding bacterial cells. (**B**) Transmission electron microscopy. TEM images of the CH-2, CH-2 ΔG148_RS04320, and cCH-2 ΔG148_RS04320 stained by phosphotungstic acid. The CH-2 and cCH-2 ΔG148_RS04320 strains were capsulated, while the CH-2 ΔG148_RS04320 strain was deficient in the ability to form a capsule.

To confirm the presence of the capsule, India ink staining and transmission electron microscopy were used to visualize the capsule on the surface of *R. anatipestifer* strains. As shown in Fig. 3A, The CH-2 and cCH-2 Δ*G148_RS04320* strains were surrounded by a clear and transparent halo, indicating the presence of a capsule, while the CH-2 Δ*G148_RS04320* strain was surrounded by a smaller halo or even no halo. TEM images also supported the presence of a capsule in CH-2 and cCH-2 Δ*G148_RS04320* strains (Fig. 3B). Conversely, no capsule was detected for CH-2 Δ*G148_RS04320*. The result indicates that *G148_RS04320* gene is involved in the capsule biosynthesis.

### LPS enables cross-agglutination of *R. anatipestifer*

**Fig 4:**
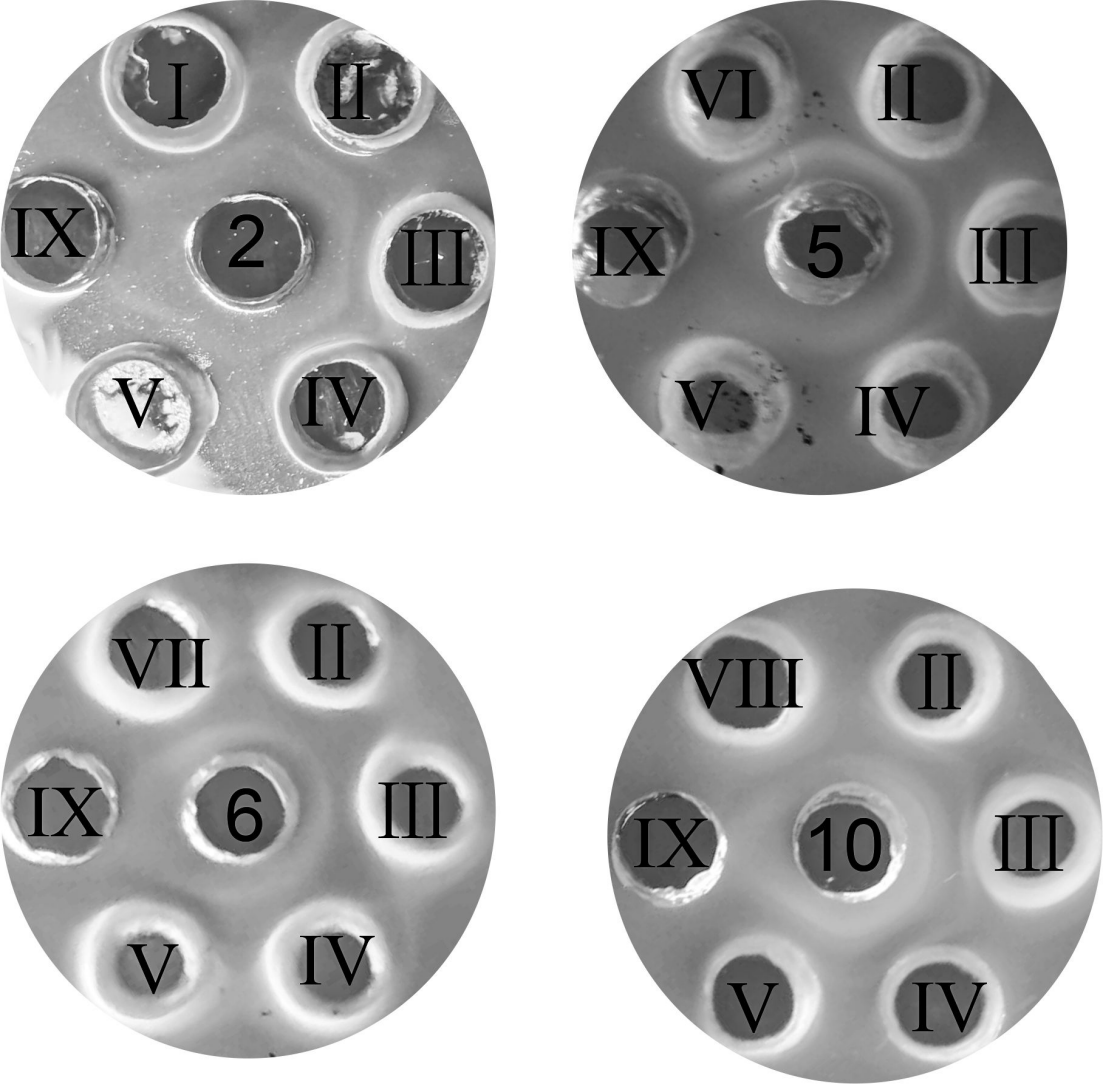
Agar-gel precipitin analysis of LPS with the antisera types 2, 5, 6, and 10. The central hole shows the standard types 2, 5, 6, and 10 antisera. The peripheral holes are labeled with roman numbers I–VII and represent LPS extractions from CCUG 25001 (serotype 2), CH-2, CH-2ΔG148_RS04320, cCH-2ΔG148_RS04320, RCAD0392, CCUG 250–04 (serotype 5), CCUG 25005 (serotype 6), and CCUG 25008 (serotype 10), respectively; hole IX shows physiological saline.

To confirm the crucial role of LPS in cross-agglutination of *R. anatipestifer*, we extracted LPS from CCUG 25001 (serovar 2), CH-2, CH-2ΔG148_RS04320, cCH2ΔG148-RS04320 and RCAD0392 and analyzed them using agar-gel precipitin test (AGPT). The results showed that antisera 2 produced a precipitation line in agarose gels with LPS extracts of these five strains (Fig. 4), indicating that LPS extracts from these five strains were capable of binding to the anti-type 2 antisera. Therefore, we suspected that LPS may be a common antigen without serovar specificity. To further confirm our hypothesis, we performed AGPT using the LPS of strains CH-2, CH-2ΔG148_RS04320, cCH2ΔG148-RS04320, RCAD0392, CCUG 25004 (serovar 5), CCUG 25005 (serovar 6), and CCUG 25008 (serovar 10) with the types 5, 6, and 10 antisera. The results demonstrated that LPS extraction from all strains produced a fused precipitation line in the agarose gel with the types 5, 6, and 10 antisera (Fig. 4; Table S2), indicating that LPS could bind to a wide range of antisera and was a common antigen rather than a specific antigen determining the serotype.

### CPS determines *R. anatipestifer* serotype

**Fig 5:**
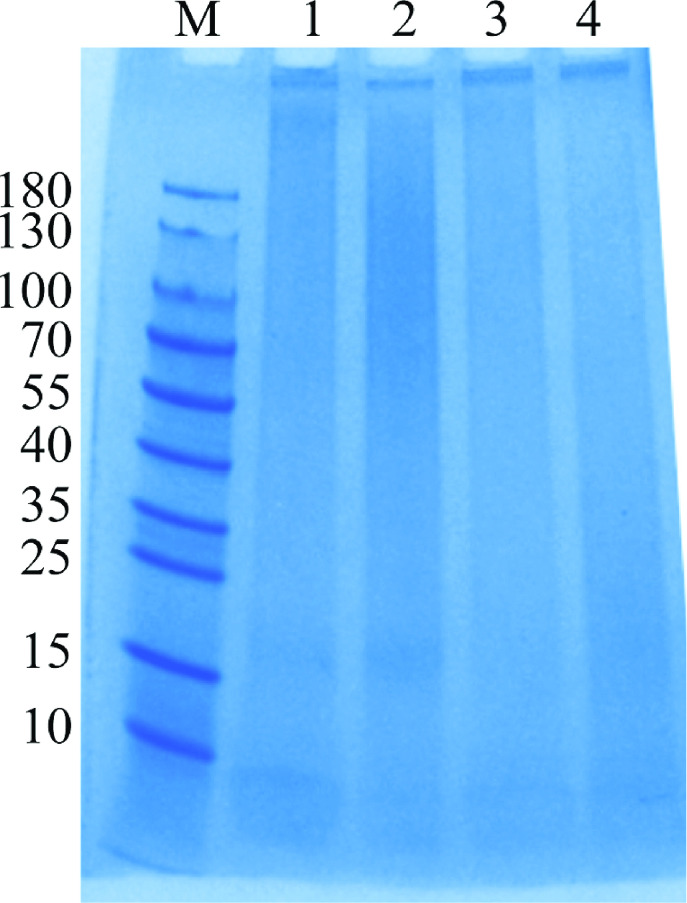
Analysis of CPS expression in *R. anatipestifer* strains. CPS was separated on a 10% SDS-PAGE and stained with Alcian blue. Line 1, CH-2; line 2, CH-2ΔG148_RS04320; line 3, cCH-2ΔG148_RS04320; line 4, RCAD0392.

**Fig 6:**
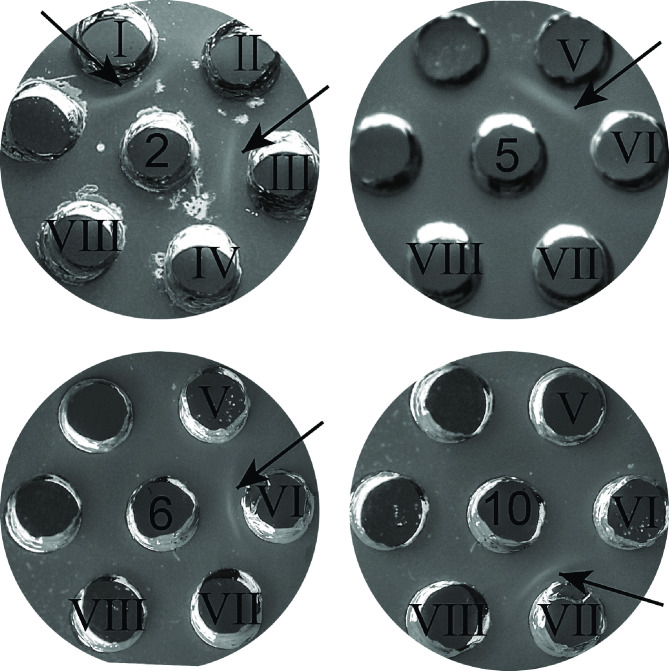
Agar-gel precipitin analysis of CPS with antisera. The central hole shows the standard types 2, 5, 6, and 10 antisera. The peripheral holes are labeled with roman numbers I–VII and represent CPS extractions from CH-2, CH-2Δ*G148_RS04320*, cCH-2Δ*G148_RS04320*, RCAD0392, CCUG 25004 (serotype 5), CCUG 25005 (serotype 6), and CCUG 25008 (serotype 10), respectively; hole VIII: physiological saline.

To validate the relationship between CPS and *R. anatipestifer* serological phenotype, CPS extracts from CH-2, CH-2ΔG148_RS04320, cCH2ΔG148-RS04320, and RCAD0392 were analyzed by SDS-PAGE and Alcian blue. As shown in Fig. 5, all four strains exhibited a capsular-like polysaccharide. The CPS of the high molecular weight is reduced in lane 2. Then, AGPT analysis of CPS extraction revealed that antisera 2 could only form a precipitation line with the CPS of CH-2, cCH-2ΔG148_RS04320 strains but not CH2ΔG148-RS04320 and RCAD0392 strains (Fig. 6). Furthermore, antisera 1 and 3–13 did not produced any precipitation lines with the CPS samples of the four stains (data not shown). These results suggested that the CPS of *R. anatipestifer* was serovar-specific and only bound to the corresponding antisera.

To further validate this finding, CPS extracts from CCUG 25004, CCUG 25005, and CCUG 25008 were analyzed using AGPT. Types 5, 6, and 10 antisera could only form a precipitation line with the CPS of CCUG 25004 (serovar 5) CCUG 25005 (serovar 6), and CCUG 25008 (serovar 10), respectively (Fig. 6). This finding supports our hypothesis that each known serotype of *R. anatipestifer* corresponds to a specific CPS.

**Fig 7:**
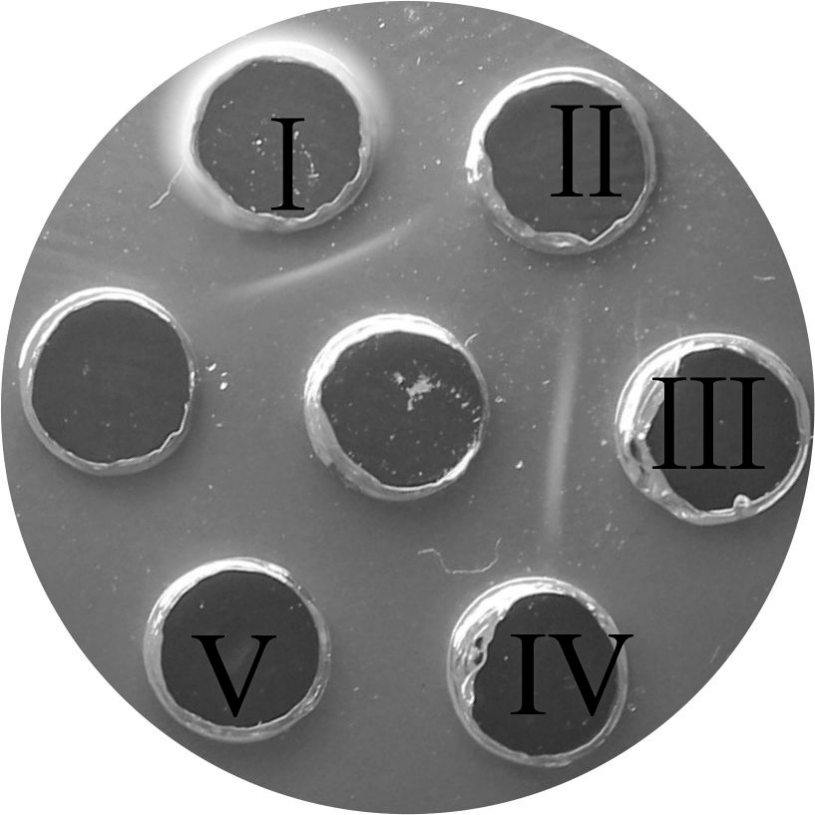
Agar-gel precipitin analysis of CPS with anti-CPS serum. The central hole shows the anti-CPS serum. The peripheral holes are labeled with roman numbers I–IV and represent CPS extractions from CH-2, CH-2Δ*G148_RS04320*, cCH-2Δ*G148_RS04320,* and RCAD0392. Hole V: physiological saline.

To confirm that CPS was the specific antigen responsible for *R. anatipestifer* serotyping, CPS from CH-2 was used to immunize mice and obtain antisera. AGPT using the obtained mouse serum was used as antibody and the CPS extracts from CH-2, CH-2ΔG148_RS04320, cCH2ΔG148-RS04320, and RCAD0392 as antigen. As expected, the antisera produced a precipitation line only with the CPS of CH-2 and cCH-2ΔG148_RS04320 (Fig. 7). These results indicated that CPS was a specific antigen that determined *R. anatipestifer* serotyping.

## DISCUSSION

As we all know, a wide variety of bacteria can be serotyped based on their polysaccharides. Currently, there are up to 21 serotypes of *R. anatipestifer,* and serotyping is still based on traditional serological agglutination tests (1). However, the exact polysaccharide(s) that determine *R. anatipestifer* serotype is still unknown (11). In this study, we determined that the CPS is the polysaccharide antigen that determines the serotyping of *R. anatipestifer*, and found that the LPS of *R. anatipestifer* mediates cross-agglutination with antisera in the absence of the capsule on the surface.

In this study, the CPS gene cluster of *R. anatipestifer* was found to be located between *recX* and *rimO* genes, and the CPS locus of different serovars were also located between this gene pair. Notably, the CPS locus of *Elizabethkingia anophelis*, another member of the *Weeksellaceae* family, is also located downstream of the conserved *recX* gene (20). This is similar to several other species where the CPS locus lies between conserved gene pairs in the genome (30). *R. anatipestifer* is generally not prone to cross-agglutination with heterologous antisera. However, in this study, we isolated a naturally occurring strain, RCAD0392, which exhibited cross-agglutination properties. Further analysis revealed that the CPS of RCAD0392 is almost identical (Identity >99%) to that of the closely related strain RA CH-2 (31), except for a frameshift mutation in the *D1J34_RS08130* gene (corresponding to *G148_RS04320* of RA CH-2), resulting in a disrupted coding region. Thus, we constructed a deletion strain of gene *G148_RS04320* (homolog of *D1J34_RS08130*) in RA CH-2, which was also able to cross-agglutinate with a variety of antisera, while the complement strain regained serological reaction specificity. This indicated that the appearance of the cross-agglutination phenotype of RA CH-2 was indeed due to the mutation in *G148_RS04320* gene. Since vaccines are only effective against homologous serotypes of *R. anatipestifer* (2), the mutations that cause the capsule defect could potentially facilitate evasion of host-specific immune stress. However, the loss of the physical barrier function of the capsule could make bacteria more susceptible to common environmental stresses, such as antibiotics.

Gene *G148_RS04320* is homologous to *gdr* in *Acinetobacter baumannii*, *cps*E in *Streptococcus suis,* and gene *pgl*F in *Campylobacter jejuni*. In *Acinetobacter baumannii* (32), *Streptococcus suis* (33), and *Campylobacter jejuni* (34), this gene encodes UDP-N-acetyl-glucosamine dehydratase, which is associated with CPS biosynthesis. Wang et al. (35) found that the bacterial morphology of the AS87_04050 mutant strain was converted from the smooth type of the wild strain to the rough type. After BLAST, the gene *AS87_04050* is also found to be on the CPS synthesis gene cluster. This represents a typical morphological alteration associated with the loss of capsule (36). Yi et al. (37) found that deletion of the gene *wza* on the RA CH-1 CPS synthesis gene cluster resulted in capsule defects. In addition, Smith et al. (38) found that the capsule was not detectable on the surface of cpsΔEF and that the parent strain agglutinated only with type 2 antisera, while the mutant strain agglutinated with all antisera. The present study observed that deletion of gene *G148_RS04320* also resulted in the disappearance of the capsule on the surface of RA CH-2, as shown by India ink staining and transmission electron microscopy. However, the complementation strain (cCH2Δ*G148-RS04320*) gave only partly phenotypic complementation, which may be due to the relatively low replication and expression levels of plasmids used for complementary strain. For the mutant strain (CH-2 Δ*G148_RS04320*), the loss of the capsule on the surface of the bacteria exposes surface antigens that would otherwise be masked by the capsule, suggesting that the agglutination of the CH-2 Δ*G148_RS04320* strain with multiple sera must be caused by its binding to polysaccharides other than CPS. This finding was consistent with previous studies (37, 38) that showed that deletion of genes in the CPS synthesis gene cluster resulted in capsule defects and the exposure of surface antigens.

We extracted LPS of CH-2, CH-2Δ*G148_RS04320*, cCH2Δ*G148-RS04320,* and RCAD0392; all of which produced precipitated lines when reacted with types 2, 5, 6, and 10 antisera. This result suggested that LPS was a common antigen rather than a specific antigen that determined serological characteristics. Yang et al. (39) found that immunizing mice with capsular reduced mutant strains can produce cross-protection against different serovars of *Pasteurella multocida*, which may be due to the effect of their surface cross antigens. Therefore, the ability of the capsule-deficient strain (CH-2Δ*G148_RS04320*) in this study to undergo cross-agglutination may be due to the exposed LPS antigens binding with multiple antisera.

We also extracted CPS of CH-2, CH-2ΔG148_RS04320, cCH2ΔG148-RS04320, and RCAD0392 and analyzed them using Alcian blue, which has been used to stain the CPS of many bacterial species (40). Although the results indicated the presence of CPS components in the mutant strain (CH-2Δ*G148_RS04320*), there was an increase in low molecular weight CPS components compared to the wild type (CH-2). Furthermore, only CPS extracts from the wild-type and complemented strains exhibited specific reactions with type 2 antisera. This suggested that the deletion of the *G148_RS04320* gene resulted in the inability of bacteria to synthesize complete CPS molecules. Instead, intermediate products of CPS synthesis accumulate within the cells, preventing the formation of a complete surface capsule structure. The CPS extracts from other strains of different serovars also showed specific reactions with their respective homologous antisera. This was further confirmed by AGPT using antisera against CPS.

In conclusion, we demonstrated that the *G148_RS04320* gene is involved in CPS biosynthesis and that the serovars of *R. anatipestifer* are distinguished by CPS. This study has provided a preliminary exploration of the molecular basis related to serotyping in *R. anatipestifer* and suggests possible directions for further investigation of the genetic basis of serotypes in this bacterium.

## SUPPLEMENTAL MATERIAL

Fig. S1 to S3 spectrum.01804-23-s0001.doc Fig. S1 (Gene cluster locus was conserved across serovars), S2 (Identification of G148_RS04320 deletion and complementation strain), and S3 (Slide agglutination of CH-2 and cCH-2ΔG148_RS04320).

Tables S1 and S2 spectrum.01804-23-s0002.xlsx Tables S1 (The variations [SNPs, INDELs, and large structural variations] between RCAD0392 and RA-CH-2) and S2 (Agar-gel precipitin analysis).

ASM does not own the copyrights to Supplemental Material that may be linked to, or accessed through, an article. The authors have granted ASM a non-exclusive, world-wide license to publish the Supplemental Material files. Please contact the corresponding author directly for reuse.
