# Supplementary material for: Capsular polysaccharide determines the serotyping of Riemerella anatipestifer
Source: Microbiol Spectr. 2023 Oct 12;11(6):e01804-23. doi: 10.1128/spectrum.01804-23 (PMC10714938; doi:10.1128/spectrum.01804-23)
Supplement: Fig. S1 to S3 — Fig. S1 (Gene cluster locus was conserved across serovars), S2 (Identification of G148_RS04320 deletion and complementation strain), and S3 (Slide agglutination of CH-2 and cCH-2ΔG148_RS04320). [file spectrum.01804-23-s0001.doc]

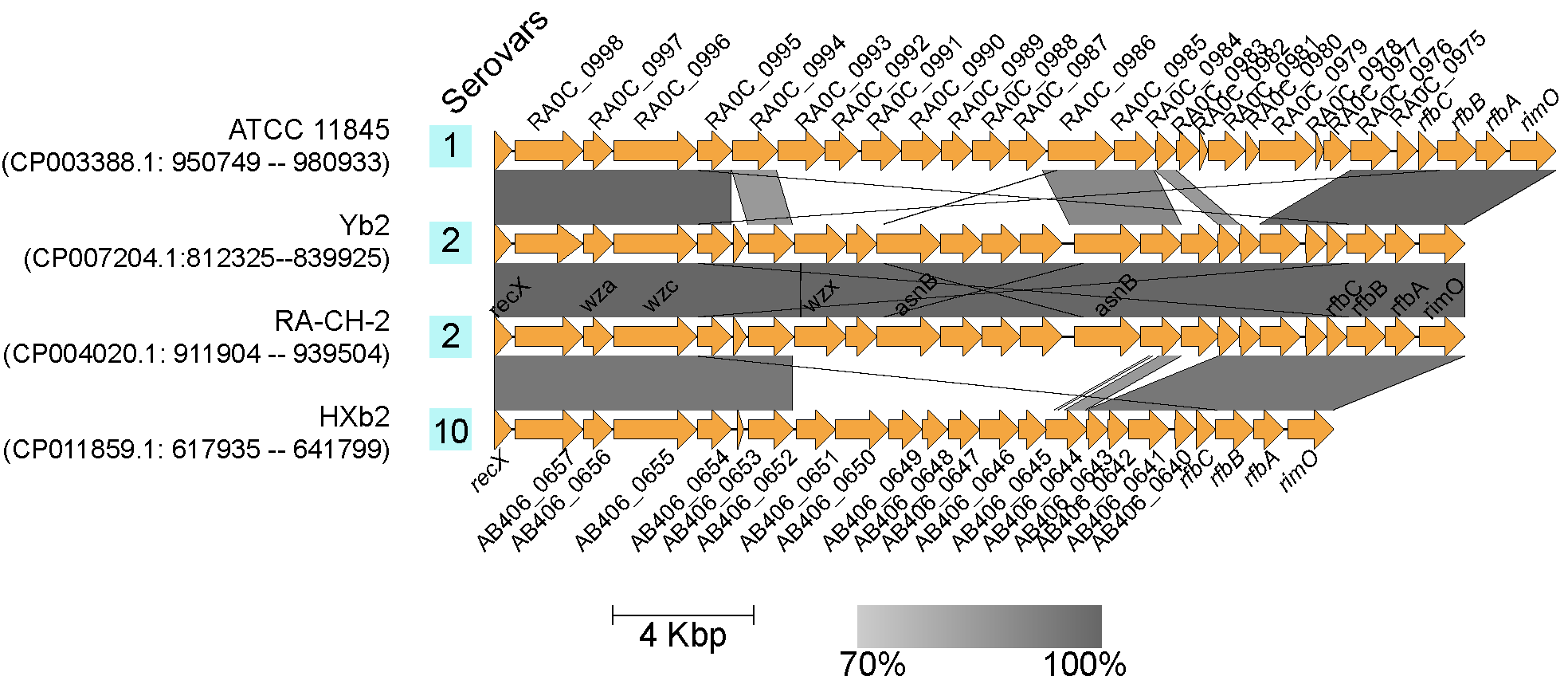


**Figure S1** Gene cluster locus was conserved across serovars

The gene annotation information is labeled based on the records from the NCBI GenBank database.


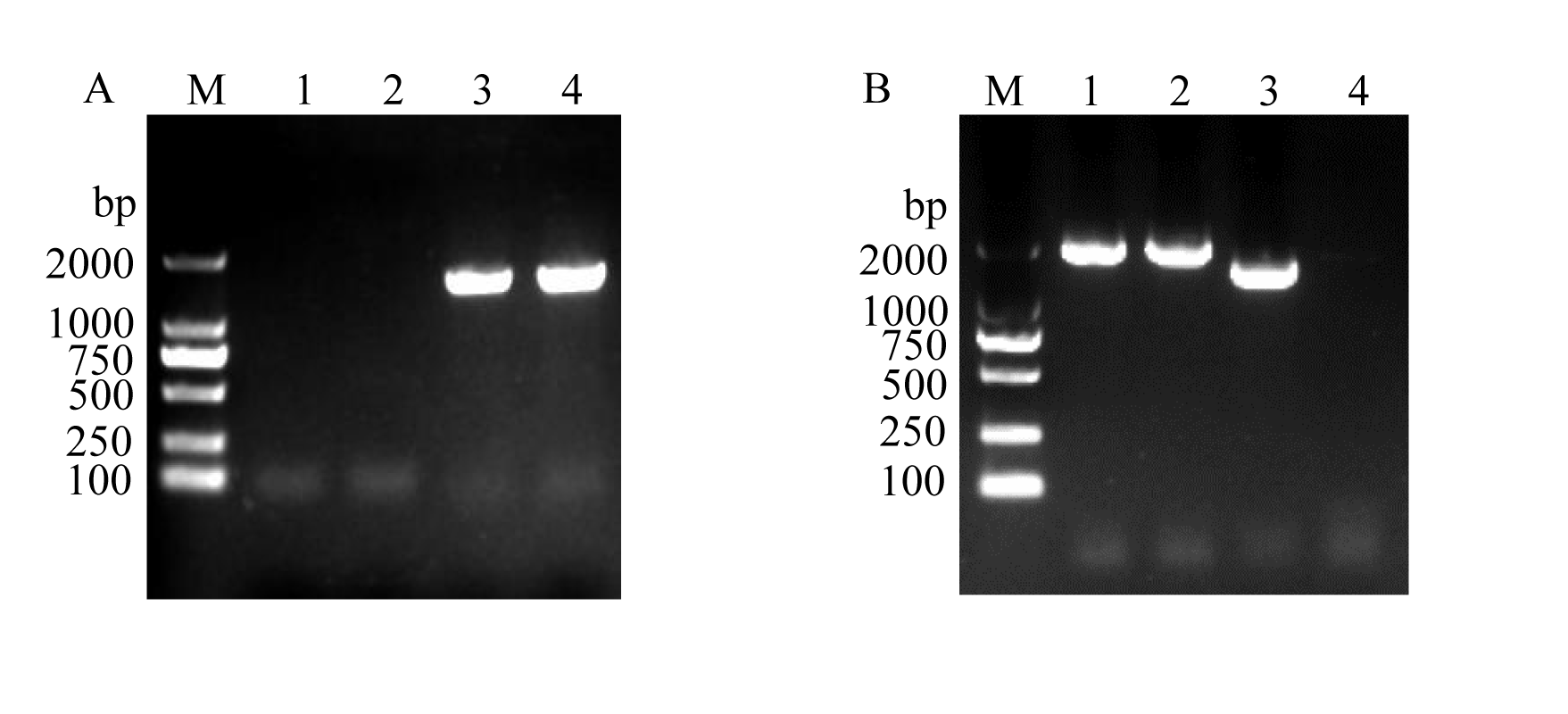


**Figure S2** Identification of *G148_RS04320* deletion and complementation strain.

(A) PCR identification of the mutant strain, line M: DL2000 DNA Marker, lines 1-4: PCR amplification results for CH-2 ΔG148_RS04320; lines 1-2: detection of the *G148_RS04320* gene using primers G148_RS04320 P1/P2; Lines 3-4: detection of the *erm* gene using primers Erm P1/P2. (B) PCR identification of the complementation strain, line M: DL2000 DNA Marker, Lines 1-3: PCR amplification results for the complementation strain, line 4: negative control; lines 1-2: detection of the *G148_RS04320* gene using primers G148_RS04320 P1/P2; Lines 3-4：detection of the *erm* gene using primers Erm P1/P2


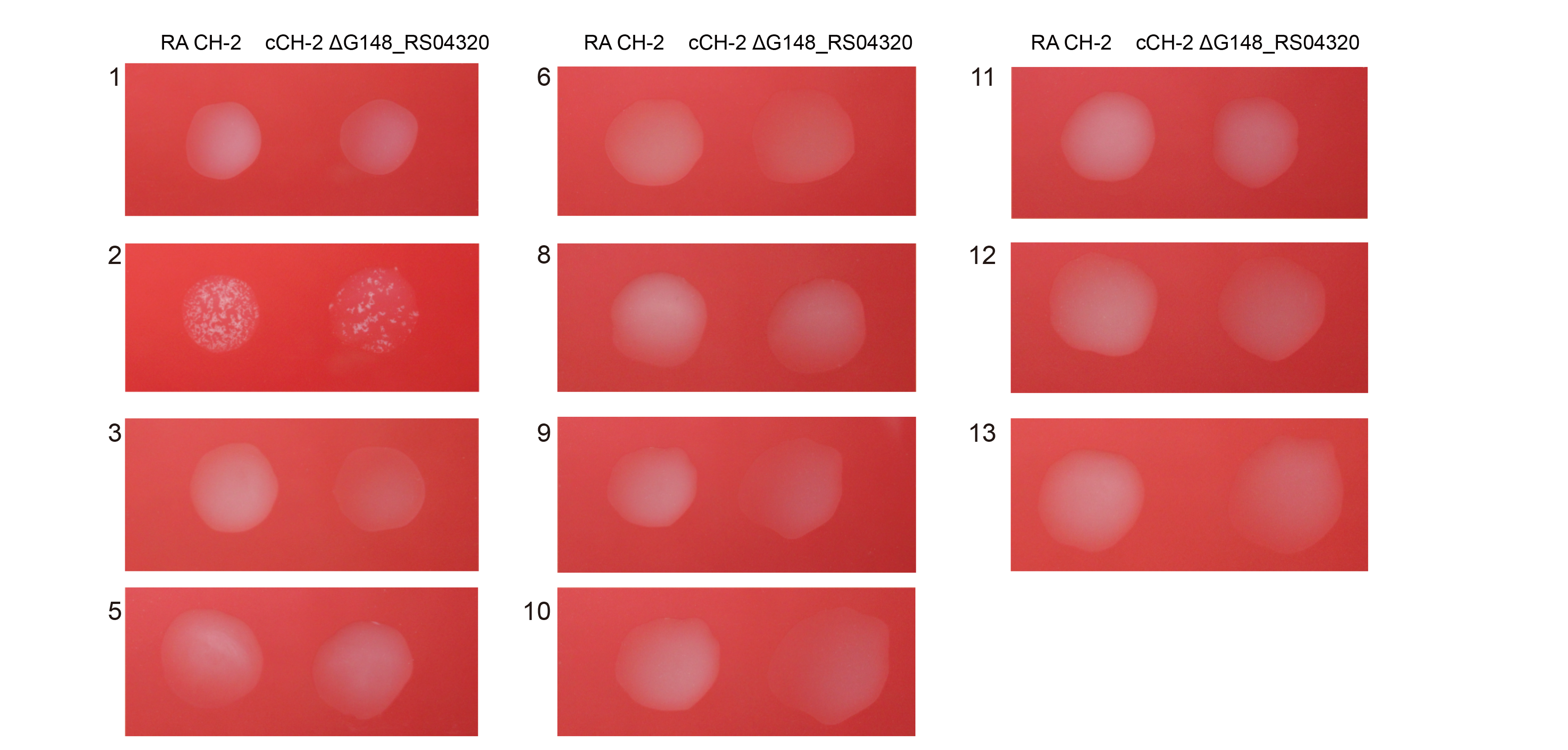


**Figure S3** Slide agglutination of CH-2 and cCH-2ΔG148_RS04320

The numbers in the top left corner of each small picture indicate standard antiserum 1-3, 5, 6 and 8-13. The CH-2 and cCH-2ΔG148_RS04320 strains could only agglutinate with serotype 2 antiserum.
